# Bat behavioral immune responses in social contexts: current knowledge and future directions

**DOI:** 10.3389/fimmu.2023.1232556

**Published:** 2023-08-17

**Authors:** Sebastian Stockmaier

**Affiliations:** ^1^ Department of Ecology and Evolutionary Biology, University of Tennessee, Knoxville, Knoxville, TN, United States; ^2^ Smithsonian Tropical Research Institute, Balboa, Ancón, Panama

**Keywords:** sickness behavior, bats (Chiroptera), inflammatory response, avoidance, infection-induced behavior, bat immunology

## Abstract

Animals often mount complex immune responses to infections. Aside from cellular and molecular defense mechanisms, animals can alter their behavior in response to infection by avoiding, resisting, or tolerating negative effects of pathogens. These behaviors are often connected to cellular and molecular immune responses. For instance, sickness behaviors are a set of behavioral changes triggered by the host inflammatory response (e.g., cytokines) and could aid in resisting or tolerating infection, as well as affect transmission dynamics if sick animals socially withdraw or are being avoided by others. To fully understand the group and population level transmission dynamics and consequences of pathogen infections in bats, it is not only important to consider cellular and molecular defense mechanisms, but also behavioral mechanisms, and how both interact. Although there has been increasing interest in bat immune responses due to their ability to successfully cope with viral infections, few studies have explored behavioral anti-pathogen defense mechanisms. My main objective is to explore the interaction of cellular and molecular defense mechanisms, and behavioral alterations that results from infection in bats, and to outline current knowledge and future research avenues in this field.

## Introduction

Animals often change their behavior in response to infection, which can have profound impacts on how individuals recover, or transmit pathogens (1–5). These behaviors can aid in avoiding, resisting, or tolerating negative fitness consequences of pathogenic infections and are often tightly connected to cellular and molecular immune responses such as inflammatory processes (2, 6, 7). For instance, sickness behaviors such as lethargy, anorexia or social withdrawal are triggered by a complex interaction of host inflammatory responses, neuroendocrinological mechanisms and the brain (1, 2, 4, 8, 9). Sickness behaviors could help the infected individual increase its tolerance to the infection (10) or resist the pathogen by diverting energetic resources to a costly immune response (5). These same behaviors can also affect uninfected conspecifics if infected individuals withdraw from social situations and, hence, are less likely to infect others (11–14). Uninfected conspecifics can also detect behavioral changes or other cues in their infected conspecifics and avoid them (3, 15).

**Figure 1:**
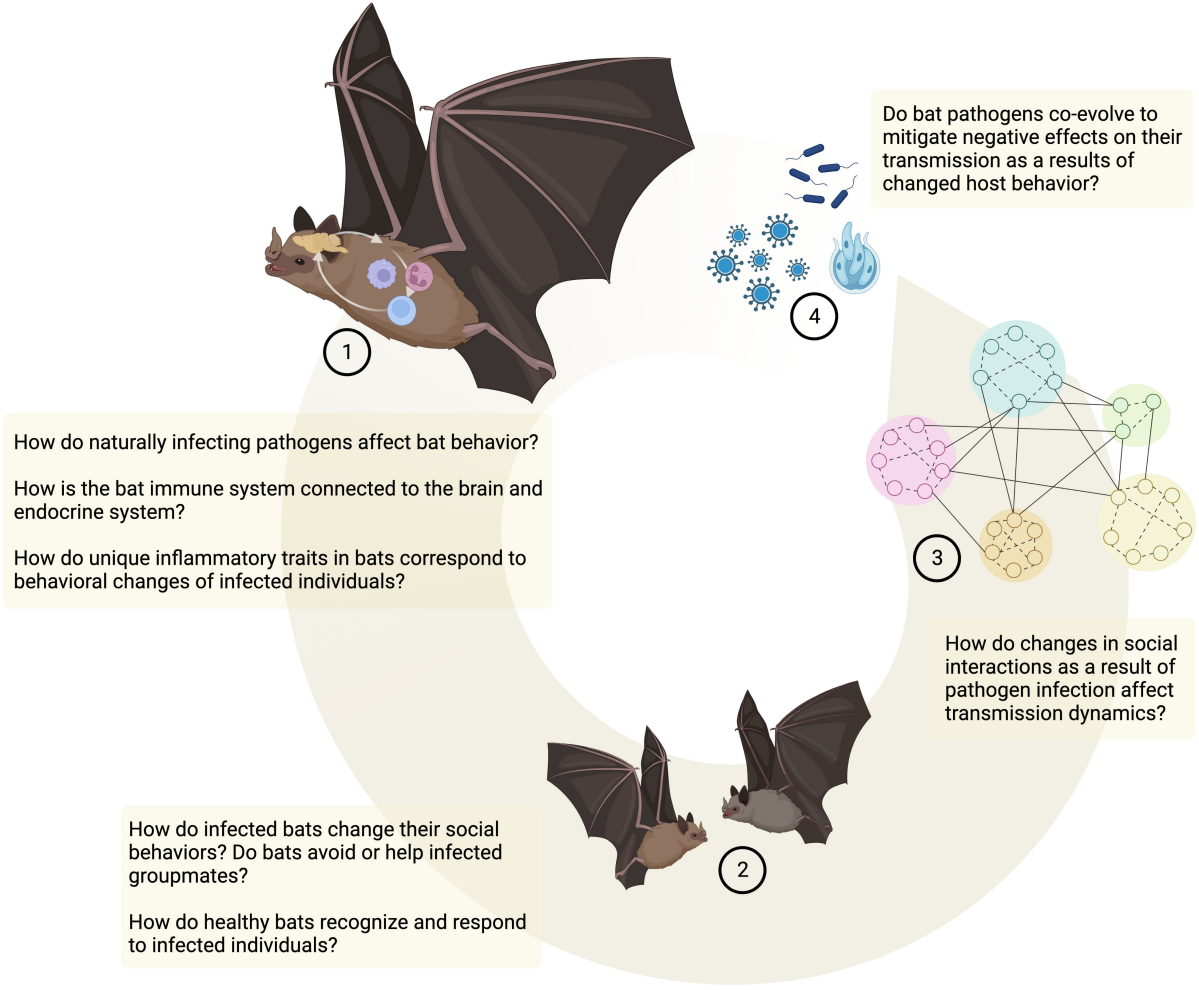
Behavioral anti-pathogen defenses in bats. Open questions and future directions. Created in BioRender.

Despite mounting interest in how bats harbor highly pathogenic viruses and what immunological mechanisms are involved in controlling viral pathogenesis (16–19), there are relatively few examples that explore behavioral anti-pathogen defense mechanisms and their connection to host immunology in bats (but see (11, 20)). Here, I will explore how infection and subsequent immune responses may lead to behavioral defense mechanisms in bats and affect their social interactions. I will mainly focus on the behavioral component of this interaction, as *in vitro* and *in vivo* studies on cellular and molecular immune responses in bats have been reviewed rigorously elsewhere (see (16, 21)). While I will outline existing studies, I will also highlight gaps in our knowledge and behavioral mechanisms to be further explored (**Figure 1**). Overall, I aim to connect research studying cellular and molecular defense mechanisms in bats and research trying to tie these immune traits to behavioral alterations that might affect downstream transmission dynamics and host-pathogen co-evolution.

## Behavioral changes as a response to infection

Upon infection with pathogens (or exposure to inflammatory triggers), the body initiates a cascade of innate responses, which lead to physiological changes in the infected animal and ultimately affect their behaviors (2, 4, 7). Conserved receptors on immune cells (pathogen recognition receptors, PRRs) recognize equally conserved pathogen-associated molecular patterns (PAMPs) and initiate inflammatory responses such as the secretion of pro-inflammatory cytokines, including Interleukin-1 beta (IL-1β), Interleukin-6 (IL-6), Tumor necrosis factor alpha (TNF-α), and Interferons such as Interferon gamma (INF-γ), among others (2, 7, 22).

Cytokine secretion not only helps the animal to reduce pathogen proliferation in early stages of the infection by protecting and preparing nearby cells, but also initiates the adaptive arm of the immune system (23, 24). Importantly, these early immune responses also change the behavior of the host. Sickness behaviors such as lethargy and social withdrawal among others, are a direct result of in increased circulation of proinflammatory cytokines acting in the periphery and in the brain (2, 4, 6–8, 22), and recent research has begun to identify specific neuronal populations that are likely involved in triggering and mediating sickness behaviors (25, 26).

While the adaptiveness of sickness behaviors is still debated (4, 27, 28), one way they could function to increase an individual’s resistance is by helping to clear the infection. For instance, sickness-induced lethargy could divert energy to metabolically costly responses such as fevers (4). Sickness behaviors could also increase tolerance of the host by promoting stress tolerance in tissues (10) or affect transmission to conspecifics if sick individual withdraw socially or move less (1, 12, 13, 27, 29).

**Figure 2:**
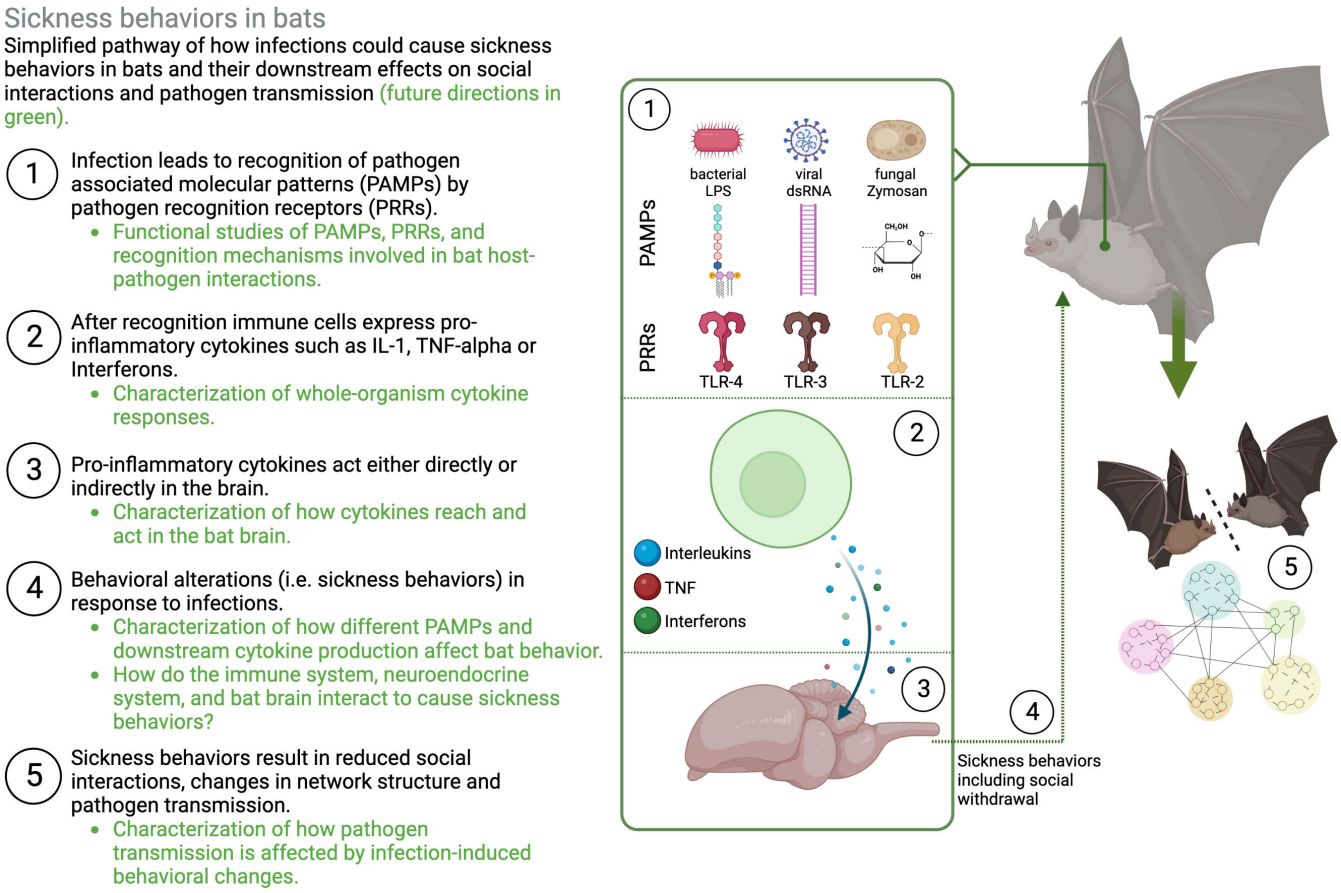
Schematic representation of sickness behaviors in bats. Illustrating the need to understand different layers of how molecular and cellular immune responses lead to changes in behavior and downstream transmission dynamics. Figure created in BioRender.

Toll-like receptors that are crucial in the recognition mechanisms that mediate sickness behaviors are highly conserved and are present throughout the animal kingdom (23). While in many cases functional studies of the specific signaling pathways are still lacking in bats (**Figure 2**, (16)), there has been increased interest in identifying bat PRRs that are involved in the recognition of pathogens (especially viral pathogens, reviewed in (16)). For instance, in the black flying fox (*Pteropus Alecto*) a full set of TLRs (1–10) transcripts has been sequenced (30), and functional studies in different bat cell cultures confirm that sensing of a PAMP, double-stranded RNA (dsRNA) and complete viruses, is likely conserved between humans and bats (31–35). *In vivo* studies that use injections of PAMPs (e.g., Lipopolysaccharide, Poly I:C, Zymogen) elicit typical innate immune responses and physiological symptoms, such as increases in white blood cell counts, weight loss, fever, and increased oxidative stress in a wide range of bat species, with intriguing species differences that need to be further explored (36–46). This suggests that bat innate immune systems are recognizing PAMPs and mounting acute phase responses. Studies on behavioral changes that accompany this acute phase response and could lead to changes in social interactions, however, are less common and limited to only a few species. Both vampire bats (*Desmodus rotundus*) and Egyptian fruit bats (*Rosettus aegyptiacus*) express sickness behaviors such as lethargy and social withdrawal in response to injections with bacterial LPS (11, 20, 47–49). Similarly, rabies-infected vampire bats show lower levels of social interactions (50) and little brown bats (*Myotis lucifugus*) are less active when infected with *Pseudogymnoascus destructans*, the fungus that causes White-nose Syndrome (51). While such studies suggest that sickness behaviors can occur during immune responses in bats, further studies across a wider range of bat species are needed to evaluate how sickness behaviors are connected to inflammatory traits in bats (**Figure 1**).

In recent years, bats have been identified as potential reservoirs for emerging viruses (52–54), which has sparked interest in what makes them immunologically unique in tolerating pathogens that are often lethal to other species (reviewed in (16–18)). Recent studies suggest that one mechanism by which bats tolerate otherwise highly pathogenic viruses is through a sophisticated regulation of the inflammatory response, which prevents damage to the host while also limiting viral propagation (18, 19, 55, 56). Bats that are experimentally infected with viruses often do not show any symptoms of illness (57–61), and both *in vitro* and *in vivo* studies suggest that inflammatory responses to viral triggers are often dampened (for detailed reviews of signaling pathways and immunological mechanisms, see (16, 21)). This raises the question of whether dampened inflammatory responses do or do not translate into behavioral symptoms.

Viral infections often result in behavioral changes in different taxa (2, 62). For example, in mice, systemic injection of Poly I:C (synthetic dsRNA, a viral mimetic) causes physiological symptoms such as elevated levels of IFN-γ and sickness behaviors (63). Similarly, TLR-3 deficient mice have attenuated sickness behaviors in response to influenza infections (64). These findings suggest that stimulation of TLR-3 and downstream cytokine expression in the periphery can change host behavior. But, what if the inflammatory response to viral antigens is uniquely regulated as described above? Do inhibited inflammatory responses found in bats translate into less severe behavioral symptoms? What are the downstream effects of inhibited inflammation and, potentially, reduced symptoms on social interactions and transmission in bats?

## Physiological and behavioral changes that lead to infection recognition

Infection can not only change the behavior of hosts but can also cause visual, auditory, or olfactory cues that may be recognized by uninfected conspecifics. Such conspecific responses can range from conspecific avoidance (3, 65, 66) to aid provided to the sick conspecific (67), which can help them tolerate negative effects of an infection (1, 67, 68).

Before even having to engage in an immunological response, hosts could benefit from avoiding infected conspecifics (1, 3, 65, 66). Such conspecific avoidance is a widely observed behavior in vertebrates (e.g (69–73)), and offers a first line of defense. Infection itself can produce a variety of cues (olfactory (69, 72, 74); visual (75, 76); auditory (47, 77); and behavioral (2, 3)) that could lead to conspecific avoidance (3). Importantly, while avoidance of sick conspecifics has been shown in many different taxa, exact mechanisms of recognition are less well understood (but see (69)). Some studies in bats have examined conspecific responses to immune-challenged individuals (20, 47, 49). In these cases, reduced contact appeared to be primarily driven by the sick individual not engaging in social interactions (e.g., through lethargy, reduced movement, or social withdrawal) and less by conspecific avoidance. Importantly, these studies used immune-challenges as opposed to naturally infecting pathogens that might produce different infection cues (e.g., visible lesions, respiratory or neurological symptoms).

Some unanswered questions remain in bats: Do bats avoid their sick conspecifics? What are the cues and the mechanisms of recognition that mediate avoidance? What is the cost-benefit balance between conspecific avoidance and forgoing beneficial social interactions (e.g., see (49))? Given that bat species vary in which sensory system is predominantly used, does infection perception occur in the predominantly used sensory modality or in a single system (e.g., olfactory perception) regardless of which sensory modality is most frequently used in other aspects of the bats’ life?

Most bat species are social (78) and their propensity to live in social groups could (i) affect their immune-system and increase resistance, and/or (ii) help them mitigate negative effects of infection (i.e., increased tolerance), Research suggests that social integration as opposed to isolation or social adversity has a range of fitness benefits and can alter the regulation of the immune response (reviewed in (79)). For instance, experimental studies in primates suggest that increased social adversity leads to increased expression of genes linked to the inflammatory response (80, 81). While research aimed at understanding the interplay of social group living and immunity is gaining traction, studies in bats are lacking. How does living in social groups affect bat health positively or negatively, and how are immunological defense mechanisms affected by bat social interactions? Because bat species vary in their level of sociality (78), this could be addressed in comparative studies evaluating immune traits in relation to sociality. Another intriguing research avenue is “immune-priming” in response to perceived infection of conspecifics (82). This anticipatory response has been observed in several taxa (82–84). For instance, in humans, perception of disease related cues triggers the immune system to mount a stronger response (83). Due to their high levels of sociality, bats are presumably often exposed to infected conspecifics. How does their immune system respond to this exposure? Similarly, social interactions with infected others (e.g., food sharing or grooming) could lead to low-dose exposure, priming the immune system for subsequent encounters with the same pathogen.

Helping infected conspecifics can increase their tolerance by maintaining their fitness despite negative effects of infections (i.e., high pathogen load). Such help could manifest in the form of food provisions, temperature regulation, or even just in the maintenance of “normal” levels of social interactions that benefit the infected individual (i.e., some individuals might not choose to avoid their infected conspecifics). Theoretically, healthy individuals might help infected individuals tolerate infections if the individual costs are not too high (e.g., low virulence, (5, 68)) or if they indirectly benefit from helping specific individuals (e.g., socially bonded partners or kin (85)). Vampire bats will share food with immune-challenged conspecifics (49), though, here, immune-challenged bats were simultaneously fasted which makes it difficult to discern the exact cause of food sharing behaviors. The question remains if and under which social circumstances bats do not avoid, or even aid their infected conspecifics, and how this affects individual tolerance of pathogens and, potentially, supports recovery.

## Group- and population-level effects of infection-induced behavioral changes

Whether individuals socially withdraw as a response to infection, avoid infected conspecifics, or increase contact with them to provide aid, will affect how pathogens spread through their social networks (1). In humans, behavioral symptoms as a results of influenza infections reduce the number of social contacts and predicted R0 (13). Similarly, immune-challenged mice move less, are less connected, and predicted pathogen transmission is therefore contained within few animals (12). While the behavioral effects of infection, or immune-challenges on individuals and their downstream effects on group characteristics and dynamics have been described in other species (12, 86, 87), this has rarely been done in bats (but see 48), especially with pathogens that infect them naturally. The question overall remains, how do sick bats and their groupmates behave, and how does this affect transmission to others? If sickness behaviors are connected to an inflammatory response, they can have downstream effects on how pathogens are transmitted illustrating the need to understand different layers of how inflammatory responses affect (i) the expression of sickness behaviors, (ii) interactions with others, and (ii) group of population-level transmission dynamics (**Figure 2**).

Many bats live in fission-fusion type social organizations or disperse, involving frequent movement of individuals to other roosts (78). How does reduced movement because of infections affect spread to other roosts and new individuals? New methods will allow for answers to some of these questions. For instance, the questions of how sick bats interact and move and how this could affect pathogen transmission to others might be answered using combinations of diagnostic methods to detect active infection in bats, and next-generation animal tracking methods such as GPS loggers and proximity sensors that quantify movement and host-host contacts (20, 48, 88).

Importantly, these infection-induced changes in behavior can also affect host-pathogen co-evolution if they affect transmission of the pathogen. Pathogens could evolve changes in virulence, pre- or asymptomatic periods, and reductions in infection-induced cues to avoid detection (1, 89, 90). The ability of viruses to mitigate sickness behaviors that might affect their transmission has recently been suggested in the case of SARS-CoV2, where a viral protein can help silence pain which could theoretically reduce sickness behaviors and increase transmission (91).

## Concluding remarks

While the unique ability to deal with certain pathogens has triggered an interest in bat's inflammatory traits (16–19, 21), there has been far less interest in how these immune responses affect bat behavior even though behavioral alterations might affect pathogen transmission and host-pathogen co-evolution (1). Future research on immunological responses in live bats, and especially studies using experimental infections with bat-infecting pathogens, should include behavioral components to answer questions of how sick bats behave and how this affects their healthy conspecifics and groups. Behavioral observations could range from scoring of individual or social behaviors (11, 50) to more sophisticated methods such as proximity sensors used to quantify individual-individual contacts and network-wide effects on transmission (48, 92), or GPS tracking to quantify bat movement in relation to their infection, or immune status (20).

Here, I argue that to understand the group- and population-level transmission dynamics and consequences of pathogenic infections in bats, it is not only important to study their molecular and cellular defense mechanisms, but also behavioral defence mechanisms, and how they interact.

## Author contributions

The author confirms being the sole contributor of this work and has approved it for publication.
